# Racial Disparities in Upper Gastrointestinal Hemorrhage Treatment

**DOI:** 10.1007/s40615-025-02335-7

**Published:** 2025-02-27

**Authors:** Riley Scherr, Jacqueline J. Chow, Caitlyn Sing, Katharine A. Kirby, Joseph A. Breuer, Nadine Abi-Jaoudeh

**Affiliations:** 1 School of Medicine, University of California Irvine, Irvine, CA USA; 2 Center for Statistical Consulting, Department of Statistics, University of California Irvine, Irvine, CA USA; 3 Department of Radiological Sciences, University of California Irvine, Orange, CA USA; 4 101 The City Dr S, Orange, CA 92868 USA

**Keywords:** Non-variceal upper GI hemorrhage, Interventional radiology, Endoscopy, Length of stay

## Abstract

**Background and Aims:**

To identify demographic predictors, with a focus on race and socioeconomic status, for advanced treatment modality, mortality, and increased length of stay (LOS) in upper gastrointestinal (GI) hemorrhage treatment.

**Methods:**

Hospitalizations with acute upper GI hemorrhage from 2016 to 2021 were identified in the Healthcare Cost and Utilization Project’s National Inpatient Sample. Cases were divided into interventional radiology (IR) and non-IR (endoscopic) treatments. Statistical analyses calculated significant odds ratios via 95% confidence intervals. The primary outcome of interest was mortality rate. The secondary outcome of interest was the mean LOS. Confounding factors affecting mortality were also examined.

**Results:**

There was no significant difference in likelihood of an IR procedure or mortality between White patients and both Non-Hispanic (NH) Black and Hispanic patients. NH Black patients had significantly longer LOS in days compared to White patients (12.61 vs 9.57) that persisted when matching for age and sex (13.78 vs 9.92), socioeconomic status (12.94 vs 10.07), chronic comorbidities (11.33 vs 8.88), blood transfusions (14.46 vs 10.21), and vasopressor use (14.43 vs 10.29) (*p* < 0.001). These LOS differences were not seen under matching conditions post-COVID-19.

**Conclusion:**

This study presents racial disparities in LOS following acute upper GI hemorrhage, but no differences in advanced treatment utilization or mortality. Confounders were responsible for LOS differences in non-IR treatment, but NH Black patients had persistently longer LOS than White patients after IR treatment.

## Introduction

Upper gastrointestinal (GI) hemorrhage is any hemorrhage of the GI system above the ligament of Treitz. Acute upper GI hemorrhage is potentially lethal and has high morbidity [1]. Odds of mortality, rebleeding, transfusion, and intervention can be calculated using a combination of the Glasgow-Blatchford and Rockall clinical scoring methods [2]. Treatments for acute upper GI hemorrhage vary and can be pharmacological coagulation, endoscopic, surgical, or angiographic. Interventional radiology’s (IR) angiographic treatment for non-variceal hemorrhage is transarterial embolization (TAE). Endoscopy is preferred first-line treatment, but TAE is often used as an alternative in unstable patients or an alternative to surgery in cases of rebleeding after endoscopy.

Racial disparities exist across a wide range of diseases and treatments. Recent papers have shown these disparities exist for other interventional radiology procedures. For example, Black and Hispanic patients are less likely to receive morbidity-reducing AV fistulas for end-stage renal disease compared to White patients [3]. Black patients were also found to have higher mortality and lower odds of undergoing IR procedures for acute pulmonary embolism [4]. Similar racial disparities in access and mortality exist for TIPS procedures and thrombectomy of ischemic stroke [5, 6].

IR treatment of acute upper GI bleeds is frequently reserved for refractory, unstable cases; access to TAE may be inequitable as it is essentially access to advanced, minimally-invasive acute care. However, there have been no studies on IR disparities in acute upper GI non-variceal hemorrhage. The limited data on racial disparities in acute GI hemorrhage show increased incidence and rebleeding in Hispanic patients, as well as greater mortality in Black patients [7, 8]. There is accordingly a need to examine the equity of TAE delivery and socioeconomic influences on outcomes, mainly mortality; that is the purpose of this study.

## Methods

### Data Source

This study was deemed exempt by the institutional review board at UCI Medical Center. Data were obtained from the National Inpatient Sample (NIS), one of the datasets available from the Healthcare Cost and Utilization Project (HCUP). The NIS is the largest publicly available inpatient database approximating a 20% stratified sample of US community hospitals, encompassing greater than 95% of the US population.

### Study Population

Data were obtained from the NIS between January 1, 2016, and December 31, 2021. 2021 was the most recent data year available. Patients were included if they met the following: hospitalization with an International Classification of Diseases, Tenth Revision, Clinical Modification (ICD-10), diagnosis code for acute upper GI hemorrhage and ICD-10 Procedure Coding System (ICD-10-PCS) procedure codes for endoscopic or TAE treatment, self-identified race as Hispanic, non-Hispanic (NH) Black, or White. GI hemorrhage codes included were K922, K2901, K31811, K27, K29, K254, K260, and K264. Endoscopic treatment was labeled non-IR and TAE treatment was labeled IR. Endoscopic ICD-10-PCS codes included were 0DQ1-9. IR ICD-10-PCS codes included were the 04L series ending in DZ. Variceal hemorrhage was excluded by confirming no TIPS (06183J4), bleeding esophageal varices (I85.01), or terlipressin administration (XW03367) codes were used. The 30233 series ending in H, J, K, L, M, N P, and R were counted as blood transfusions. Vasopressor use was identified as 3E033XZ.

### Statistical Analysis

Statistical analysis was performed utilizing SPSS 28 and Microsoft Excel for the Mac software. Significance was declared at alpha of 0.05 and determined via chi-squared test and logistic regression for demographic data. Two-tailed *t*-tests were used for length of stay. For all odds ratios, significance was determined via 95% confidence intervals.

### Case–Control Match Analysis

A propensity value to undergo IR or non-IR treatment was calculated for all patients based on demographic factors including (1) age and sex (BIO group), (2) age, sex, and socioeconomic status (SES group), and (3) age, sex, and health status (COMRB group). Median zip code income quartile and insurance type approximated socioeconomic status. Charlson comorbidity index score approximated health status. Patients of White, NH Black, and Hispanic races were then matched based on propensity scores. A patient was included in the final matched analyses if they had a propensity value matching a patient in both other races. Matched patients were then analyzed for odds of undergoing an IR intervention, odds of mortality, and LOS.

After initial analyses, propensity score matching was done between just White and NH Black patients based on (1) age, sex, and number of blood transfusions and (2) age, sex, and vasopressor use to analyze LOS differences.

## Results

### Patient Demographics

A total of 10,840 patients met inclusion criteria. Patients were divided into years before and after the COVID-19 pandemic: 8002 from 2016 to 2019, and 2838 from 2020 to 2021.

Demographic data is summarized in Table 1. A total of 8503 (78.4%) identified as White, 1453 (13.4%) as non-Hispanic (NH) Black, and 884 (8.2%) as Hispanic.

**Table 1:** Demographic data

		White + Black + Hispanic	White	Black	Hispanic
	Total count	10,840	8503	1453	884
	2020–2021	2838	2255	369	214
	2016–2019	8002	6248	1084	670
Age	Age (95% CI)	68.15 (68.42–67.87)	69.40 (69.10–69.70)	63.40 (62.63–64.17)	63.92 (62.83–65.02)
	2020–2021	67.76 (67.24–68.29)	68.85 (68.28–69.41)	63.64 (62.11–65.17)	63.45 (61.31–65.60)
	2016–2019	68.62 (68.30–68.94)	69.60 (69.25–69.95)	63.32 (62.43–64.21)	64.07 (62.80–65.34)
	Age SD	14.59	14.06	14.94	16.61
	2020–2021	14.27	13.76	15.03	16.01
	2016–2019	14.48	14.16	14.92	16.81
Gender	Male (%)	6598 (60.87%)	5164 (60.73%)	871 (59.94%)	563 (63.69%)
	2020–2021	1777 (63.61%)	1405 (62.31%)	236 (63.96%)	136 (63.55%)
	2016–2019	4821 (60.25%)	3759 (60.16%)	635 (58.58%)	427 (63.73%)
	Female (%)	4242 (39.13%)	3339 (39.27%)	582 (40.06%)	321 (36.31%)
	2020–2021	1061 (37.39%)	850 (37.69%)	133 (36.04%)	78 (36.45%)
	2016–2019	3181 (39.75%)	2489 (39.84%)	449 (41.42%)	243 (36.27%)
Payment	Medicare	7064	5761	834	469
	2020–2021	1788	1470	205	113
	2016–2019	5276	4291	629	356
	Medicaid	1065	644	261	160
	2020–2021	310	209	56	45
	2016–2019	755	435	205	115
	Private	2045	1623	248	174
	2020–2021	553	432	77	44
	2016–2019	1492	1191	171	130
	Self-pay	338	225	62	51
	2020–2021	101	74	19	8
	2016–2019	237	151	43	43
	Other (including unknown/missing)	328	250	48	30
	2020–2021	86	70	12	4
	2016–2019	242	180	36	26
Income	ZIP code income 1	2995	2010	667	318
	2020–2021	794	640	80	74
	2016–2019	2201	1370	587	244
	ZIP code income 2	2860	2356	294	210
	2020–2021	745	606	80	59
	2016–2019	2115	1750	214	151
	ZIP code income 3	2609	2124	273	212
	2020–2021	703	547	107	49
	2016–2019	1906	1577	166	163
	ZIP code income 4	2193	1878	190	125
	2020–2021	553	433	91	29
	2016–2019	1640	1445	99	96
	Unknown/missing	183	135	29	19
	2020–2021	43	29	11	3
	2016–2019	140	106	18	16
Region	New England (1)	563	493	35	35
	2020–2021	122	107	7	8
	2016–2019	441	386	28	27
	MidAtlantic (2)	1432	1126	210	96
	2020–2021	315	254	44	17
	2016–2019	1117	872	166	79
	East N. Central (3)	1779	1510	227	42
	2020–2021	422	368	43	11
	2016–2019	1357	1142	184	31
	West N. Central (4)	730	663	62	5
	2020–2021	208	191	16	1
	2016–2019	522	472	46	4
	South Atlantic (5)	2347	1711	486	150
	2020–2021	668	502	124	42
	2016–2019	1679	1209	362	108
	East S. Central (6)	794	661	126	7
	2020–2021	252	203	47	2
	2016–2019	542	458	79	5
	W. S. Central (7)	1018	670	178	170
	2020–2021	242	158	52	32
	2016–2019	776	512	126	138
	Mount. (8)	736	618	34	84
	2020–2021	229	204	4	21
	2016–2019	507	414	30	63
	Pacific (9)	1441	1051	95	295
	2020–2021	380	268	32	80
	2016–2019	1061	783	63	215
Population density	> 1 million (1 + 2)	6243	4602	1029	612
	2020–2021	1437	1057	240	140
	2016–2019	4806	3545	789	472
	250,000–999,999 (3)	2213	1799	233	181
	2020–2021	706	578	75	53
	2016–2019	1507	1221	158	128
	50,000–249,000 (4)	882	774	69	39
	2020–2021	259	230	19	10
	2016–2019	623	544	50	29
	Micropolitan (5)	836	753	53	30
	2020–2021	238	217	17	4
	2016–2019	598	536	36	26
	Neither (6)	631	558	59	14
	2020–2021	186	167	15	4
	2016–2019	445	391	44	10
	Unknown/missing	35	17	10	8
	2020–2021	12	6	3	3
	2016–2019	23	11	7	5

Mean age for all subjects was 68.15 years (Table 1). White patients were on average older than NH Black and Hispanic patients (69.40 vs 63.40 vs 63.92). With regard to sex, White, NH Black, and Hispanic patients identified as male with 60.73%, 59.94%, and 63.69% respectively.

After propensity matching on age and sex, we maintained a cohort of 4195 patients that identified as the following: 3790 White (90.3%), 237 NH Black (5.6%), and 168 Hispanic (4.0%). When propensity matching for age, sex, primary insurance type, and ZIP code median income quartile, a total of 2,850 patients remained: 2018 White (70.8%), 481 NH Black (16.9%), and 351 Hispanic (12.3%). When propensity matching for age, sex, and Charlson comorbidity index score, a total of 2050 patients remained: 1378 White (67.2%), 375 NH Black (18.3%), and 297 Hispanic (14.5%). The Charlson index score was used because the dataset lacked the variables needed to calculate the Elixhauser comorbidity index.

Propensity score matching between just White and NH Black patients on age, sex, and blood transfusions left 5888 White patients and 1391 NH Black patients. Matching between just White and NH Black patients on age, sex, and vasopressor use left 6441 White patients and 1435 NH Black patients.

### Geographic Location and ZIP Code Median Income Quartile

Region 4 (West North Central) had the Whitest patients relative to non-White patients (90.8%). Regions 5 (South Atlantic) and 9 (Pacific) had the most NH Black and Hispanic patients respectively (40.3%, 20.5%).

For the ZIP code median income quartile, the highest quartile (4) had the highest proportion of White patients (85.6%), while the lowest quartile (1) had the highest proportion of NH Black and Hispanic patients (22.3%, 10.6%).

NH Black patients had the greatest proportion living in counties with a population greater than 1 million (16.5%), whereas White patients had the greatest proportion living in micropolitan counties (90.1%).

Medicare was the most frequent insurance type (65.2%), followed by private insurance and Medicaid. White patients had a higher use of Medicare (67.8%) compared to NH Black and Hispanic patients (57.4%, 53.1%). Private insurance and Medicaid use were similar across White, NH Black, and Hispanic patients.

### Odds Ratio of Treatment and Mortality for Race and Treatment Type

There was no overall difference in mortality based on race (Table 2), which persisted when examining IR and non-IR treatments separately. All patients undergoing IR treatment in the 2016–2021 and 2016–2019 groups had an odds ratio of mortality of 2.29 and 2.21 (1.97–2.67; *p* < 0.0001: 1.84–2.64; *p* < 0.0001) (RR [95% CI]; *p*-value) compared to non-IR treatment. There was no difference in mortality odds between IR and non-IR treatment from 2020 to 2021.

**Table 2:** Mortality odds ratios

			OR	Lower CI	Upper CI	*P* value
Age	0–17		0	0	0	0
		2020–2021	0	0	0	0
		2016–2019	0	0	0	0
	18–24		0	0	0	0
		2020–2021	0	0	0	0
		2016–2019	0	0	0	0
	25–34		**0.40969648**	**0.18987602**	**0.88400423**	**0.0227721**
		2020–2021	0.65842246	0.25567239	1.69560797	0.3934646525
		2016–2019	**0.1969425**	**0.04813383**	**0.80580225**	**0.02360873**
	35–44		1.03418668	0.7010678	1.52559009	0.8750611
		2020–2021	1.03016895	0.54755331	1.93816389	0.93274405
		2016–2019	1.004672897	0.612750393	1.647273737	0.986697148
	45–54		0.98398099	0.74253838	1.30393069	0.91771595
		2020–2021	1.47252525	0.95250131	2.27645947	0.08132113
		2016–2019	0.75803555	0.51984088	1.10537265	0.15046385
	55–64		REF	REF	REF	REF
		2020–2021	REF	REF	REF	REF
		2016–2019	REF	REF	REF	REF
	65–74		1.10301521	0.90055653	1.35098964	0.34887382
		2020–2021	1.34482184	0.9767312	1.85163102	0.06902226
		2016–2019	0.93763167	0.72090556	1.21951222	0.64388813
	75–84		**1.257115385**	**1.026145449**	**1.540073186**	**0.026936665**
		2020–2021	1.282886168	0.920261301	1.78840175	0.141931906
		2016–2019	1.22608085	0.94986035	1.58262659	0.14155848
	> 85		1.26241384	0.99720293	1.59815886	0.05239222
		2020–2021	1.33871942	0.89083778	2.01178007	0.16101913
		2016–2019	1.26749017	0.94915661	1.69258826	0.10804227
Gender	Male		REF	REF	REF	REF
		2020–2021	REF	REF	REF	REF
		2016–2019	REF	REF	REF	REF
	Female		**0.82948774**	**0.72058583**	**0.95484795**	**0.00922137**
		2020–2021	0.87961413	0.70273221	1.10101828	0.265906276
		2016–2019	**0.80829928**	**0.67435275**	**0.96885157**	**0.02115548**
Race	White		REF	REF	REF	REF
		2020–2021	REF	REF	REF	REF
		2016–2019	REF	REF	REF	REF
	Black		0.99423642	0.81475528	1.21325516	0.95872701
		2020–2021	1.0081827	0.7327631	1.38712275	0.96373924
		2016–2019	1.00036681	0.77337773	1.29397798	0.99799613
	Hispanic		1.029123499	0.805889815	1.314193523	0.82942673
		2020–2021	0.98850679	0.656389665	1.488667063	0.9598768008
		2016–2019	1.088016941	0.8001997	1.479356794	0.602721586
Treatment	IR		**2.29374407**	**1.97275275**	**2.66696467**	** < 0.0001**
		2020–2021	1.1500766	0.8252813	1.60269739	0.41650838
		2016–2019	**2.20803249**	**1.84412091**	**2.64375695**	** < 0.0001**
	Non-IR		REF	REF	REF	REF
		2020–2021	REF	REF	REF	REF
		2016–2019	REF	REF	REF	REF
Payment	Medicare		**1.27276521**	**1.05645268**	**1.53336852**	**0.0111209**
		2020–2021	1.22747134	0.91625161	1.64440188	0.17032289
		2016–2019	**1.33692417**	**1.04736627**	**1.70653407**	**0.01957902**
	Medicaid		1.017837962	0.765315811	1.353681842	0.910936663
		2020–2021	1.062601133	0.692641915	1.630165809	0.793295887
		2016–2019	0.949812495	0.644458011	1.399848803	0.806756939
	Private		REF	REF	REF	REF
		2020–2021	REF	REF	REF	REF
		2016–2019	REF	REF	REF	REF
	Self-pay		**1.64311879**	**1.13466283**	**2.37941993**	**0.00856942**
		2020–2021	**2.05321782**	**1.21203922**	**3.47819059**	**0.0074845**
		2016–2019	1.21030959	0.69576072	2.10539238	0.50941858
	Other (including unknown/missing)		0.80427659	0.49137746	1.31642348	0.39317228
		2020–2021	0.80427659	0.49137746	1.31642348	0.39317228
		2016–2019	0.740814498	0.378844478	1.448631698	0.387325076
Income	ZIP code income 1		0.93518723	0.76866416	1.13778579	0.5133293
		2020–2021	1.02588672	0.74683544	1.40920409	0.88383
		2016–2019	0.86276781	0.67047251	1.11021447	0.25404344
	ZIP code income 2		0.96565613	0.79305064	1.175828773	0.741014526
		2020–2021	1.01447617	0.734892824	1.400424477	0.936296019
		2016–2019	0.925306193	0.720034822	1.189097422	0.555419617
	ZIP code income 3		0.96080825	0.78573945	1.17488372	0.71002528
		2020–2021	1.04175157	0.75285815	1.44150172	0.8168205
		2016–2019	0.88834303	0.68552684	1.1511633	0.37699897
	ZIP code income 4		REF	REF	REF	REF
		2020–2021	REF	REF	REF	REF
		2016–2019	REF	REF	REF	REF
	Unknown/missing		1.16710102	0.70984583	1.91890231	0.55373641
		2020–2021	1.21653048	0.52213104	2.83443483	0.66268048
		2016–2019	1.16103896	0.62456171	2.1583319	0.6497829
Region	New England (1)		REF	REF	REF	REF
		2020–2021	REF	REF	REF	REF
		2016–2019	REF	REF	REF	REF
	MidAtlantic (2)		1.01577391	0.723742	1.4256415	0.93398721
		2020–2021	1.32789116	0.70305094	2.50806139	0.3888585
		2016–2019	0.87273241	0.58495194	1.30209307	0.5152381
	East N. Central (3)		0.755701044	0.537057352	1.063357694	0.107800933
		2020–2021	1.032498307	0.549800104	1.938982452	0.92733982
		2016–2019	0.615754567	0.409208958	0.926552753	0.019877514
	West N. Central (4)		1.01277603	0.69116069	1.4840475	0.95269723
		2020–2021	1.46634615	0.75426683	2.85067692	0.26211079
		2016–2019	0.733666062	0.451869709	1.191197106	0.212128178
	South Atlantic (5)		0.96068376	0.69643385	1.32519877	0.81866547
		2020–2021	1.1349444	0.62248756	2.06927636	0.69269483
		2016–2019	0.85708912	0.58637841	1.25277763	0.43398941
	East S. Central (6)		0.99925915	0.68596267	1.45564605	0.99722906
		2020–2021	1.21031746	0.62468676	2.34496463	0.58350434
		2016–2019	0.79224121	0.49454196	1.26914639	0.33795058
	W. S. Central (7)		1.08460707	0.7611111	1.54559892	0.6661284
		2020–2021	1.15708358	0.59231008	2.26037418	0.68244789
		2016–2019	1.03191129	0.68181423	1.56177571	0.89073631
	Mount. (8)		0.808503836	0.542216087	1.205568166	0.30115653
		2020–2021	1.027448534	0.517155197	2.041264396	0.943726166
		2016–2019	0.618031766	0.370830225	1.03002193	0.064421301
	Pacific (9)		0.98648814	0.70220339	1.38586465	0.94289472
		2020–2021	1.39887218	0.75215765	2.60163994	0.29289015
		2016–2019	0.743786894	0.491885094	1.124691419	0.161242767
Population density	> 1 million (1 + 2)		REF	REF	REF	REF
		2020–2021	REF	REF	REF	REF
		2016–2019	REF	REF	REF	REF
	250,000–999,999 (3)		1.00352487	0.8443168	1.19275392	0.97113665
		2020–2021	0.99301691	0.76285183	1.29262663	0.9622547471
		2016–2019	0.895004598	0.708156301	1.131153151	0.359034006
	50,000–249,000 (4)		0.914385634	0.704150246	1.187390181	0.512224724
		2020–2021	0.882031482	0.588628755	1.321681159	0.5542511472
		2016–2019	0.863897878	0.611134031	1.221204362	0.415000427
	Micropolitan (5)		0.83711697	0.63447185	1.10448529	0.21034663
		2020–2021	0.89793148	0.59192997	1.36212218	0.6251523582
		2016–2019	0.73200157	0.50162733	1.06817605	0.10549711
	None of the above (6)		1.29133044	0.99832252	1.67033626	0.05112536
		2020–2021	1.45151515	0.99125792	2.12547733	0.05511497
		2016–2019	1.04188843	0.71876584	1.5102714	0.83957928

There was no significant difference in likelihood of receiving IR treatment based on race (Table 3).

**Table 3:** Odds ratios

			OR	Lower CI	Upper CI	*P* value
Unmatched	White		REF	REF	REF	REF
		2020–2021	REF	REF	REF	REF
		2016–2019	REF	REF	REF	REF
	Black		0.9962396	0.89085264	1.11409373	0.9520212355
		2020–2021	0.74794361	0.55123962	1.01483931	0.06172731684
		2016–2019	1.0733492	0.94276018	1.22202712	0.28863869
	Hispanic		0.877602078	0.763970107	1.008135528	0.064570335
		2020–2021	0.840327483	0.562914062	1.254454856	0.4019428838
		2016–2019	0.910419989	0.774298209	1.070472006	0.258966611
PROP. sex + age	White		REF	REF	REF	REF
		2020–2021	REF	REF	REF	REF
		2016–2019	REF	REF	REF	REF
	Black		**0.59823502**	**0.50432191**	**0.70963631**	** < 0.0001**
		2020–2021	**0.5282203**	**0.33077342**	**0.84352814**	**0.007540791358**
		2016–2019	**0.60971954**	**0.50296349**	**0.739135**	** < 0.0001**
	Hispanic		1.029123499	0.805889815	1.314193523	0.82942673
		2020–2021	0.98850679	0.656389665	1.488667063	0.9598768008
		2016–2019	1.088016941	0.8001997	1.479356794	0.602721586
PROP. sex, age, zip, INS	White		REF	REF	REF	REF
		2020–2021	REF	REF	REF	REF
		2016–2019	REF	REF	REF	REF
	Black		**0.63909667**	**0.51548111**	**0.79235602**	** < 0.0001**
		2020–2021	0.58321678	0.29602179	1.14904317	0.1191021228
		2016–2019	**0.68068734**	**0.53260762**	**0.86993734**	**0.00217112**
	Hispanic		**0.596035587**	**0.46805933**	**0.759002968**	** < 0.0001**
		2020–2021	0.872727273	0.35895738	2.121847706	0.7765773108
		2016–2019	**0.593869732**	**0.449974616**	**0.783780343**	**0.000254614**
PROP. sex, age, comorb	White		REF	REF	REF	REF
		2020–2021	REF	REF	REF	REF
		2016–2019	REF	REF	REF	REF
	Black		**0.71410373**	**0.5653987**	**0.90191955**	**0.004747491421**
		2020–2021	1.84150327	0.60720265	5.5848476	0.28438937
		2016–2019	**0.64786179**	**0.50550132**	**0.83031415**	**0.00064295**
	Hispanic		**0.625586354**	**0.485118223**	**0.806727655**	**0.0003256777799**
		2020–2021	0.952069717	0.36229627	2.501921273	0.9272133106
		2016–2019	**0.557914747**	**0.422120532**	**0.73739333**	** < 0.0001**

### Other Sociodemographic Predictors of Mortality

Females had a 0.83 (0.72–0.95; *p* < 0.01) (RR [95% CI]; *p*-value) odds of mortality compared to males. Age had little effect on the relative odds of mortality, with only ages 25–34 years associated with a decreased odds of mortality relative to ages 55–64 years (0.41, 0.19–0.88; *p* = 0.02) (RR, 95% CI; *p*-value). Patients in the oldest age groups had increased mortality odds (1.26, 1.03 – 1.54; *p* = 0.03) (RR, 95% CI; *p*-value). Medicare and self-pay status were associated with greater mortality odds (1.27, 1.05 – 1.53; *p* = 0.01: 1.64, 1.13 – 2.38; *p* < 0.01) (RR, 95% CI; *p*-value). Geographic region, population density, and ZIP code median income quartile were not associated with a significant mortality risk.

### Race and Length of Stay

**Fig. 1:**
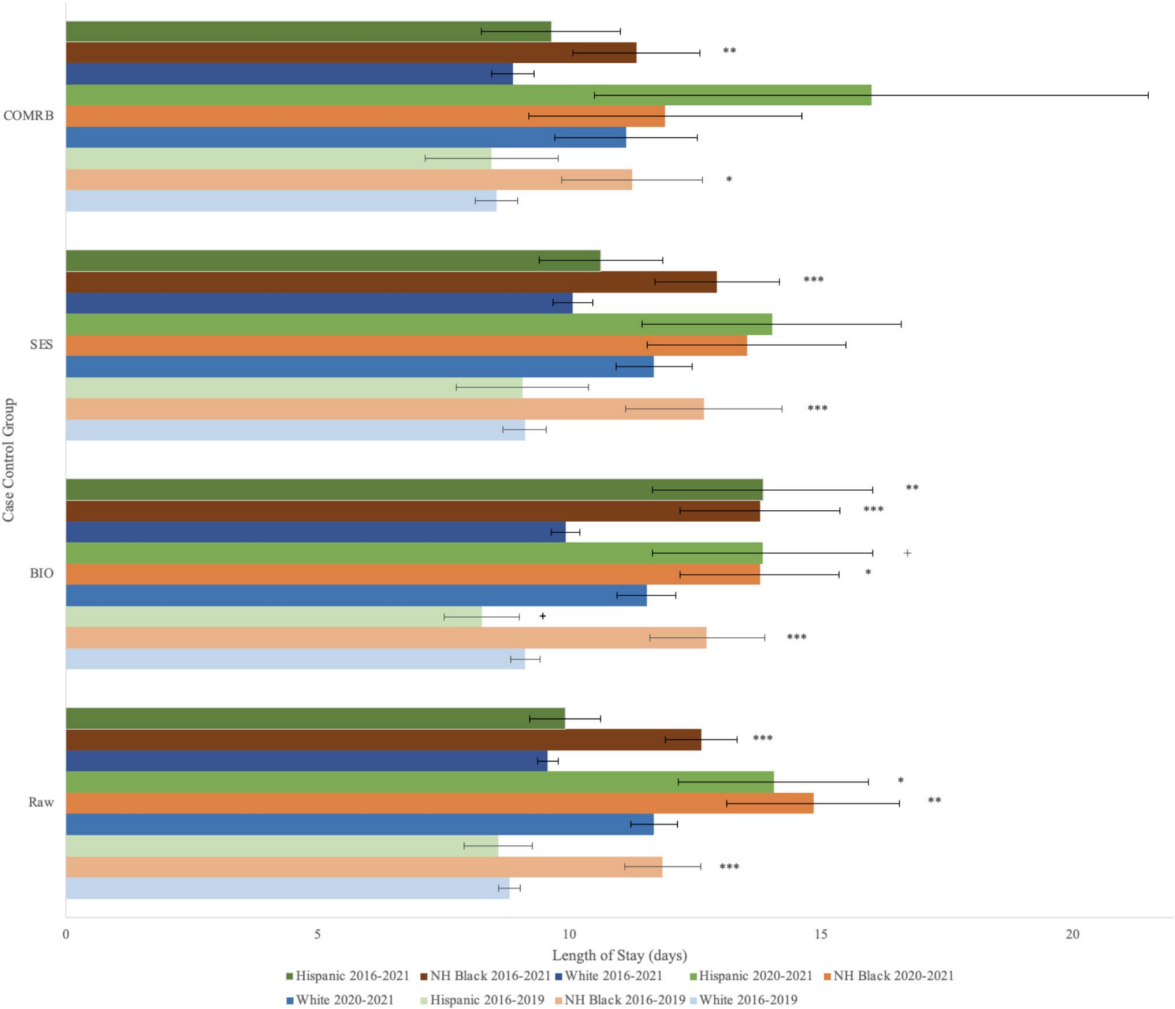
Overall length of stay regardless of treatment type for case-controlled datasets. Length of stay by self-reported race for patients treated with both IR and non-IR treatment for acute upper non-variceal GI hemorrhage. Error bars indicate 95% confidence intervals. Significance between non-White race and White race determined by two-tailed *t-*test. *** indicates *p* < 0.0001, ** indicates *p* < 0.001, * indicates *p* < 0.01, ^+^ indicates *p* < 0.05. Raw, uncontrolled dataset; BIO, controlled for age and biological sex; SES, controlled for age, biological sex, median ZIP code income quartile, and primary insurance type; COMRB, controlled for age, biological sex, and Charlson comorbidity index

**Fig. 2:**
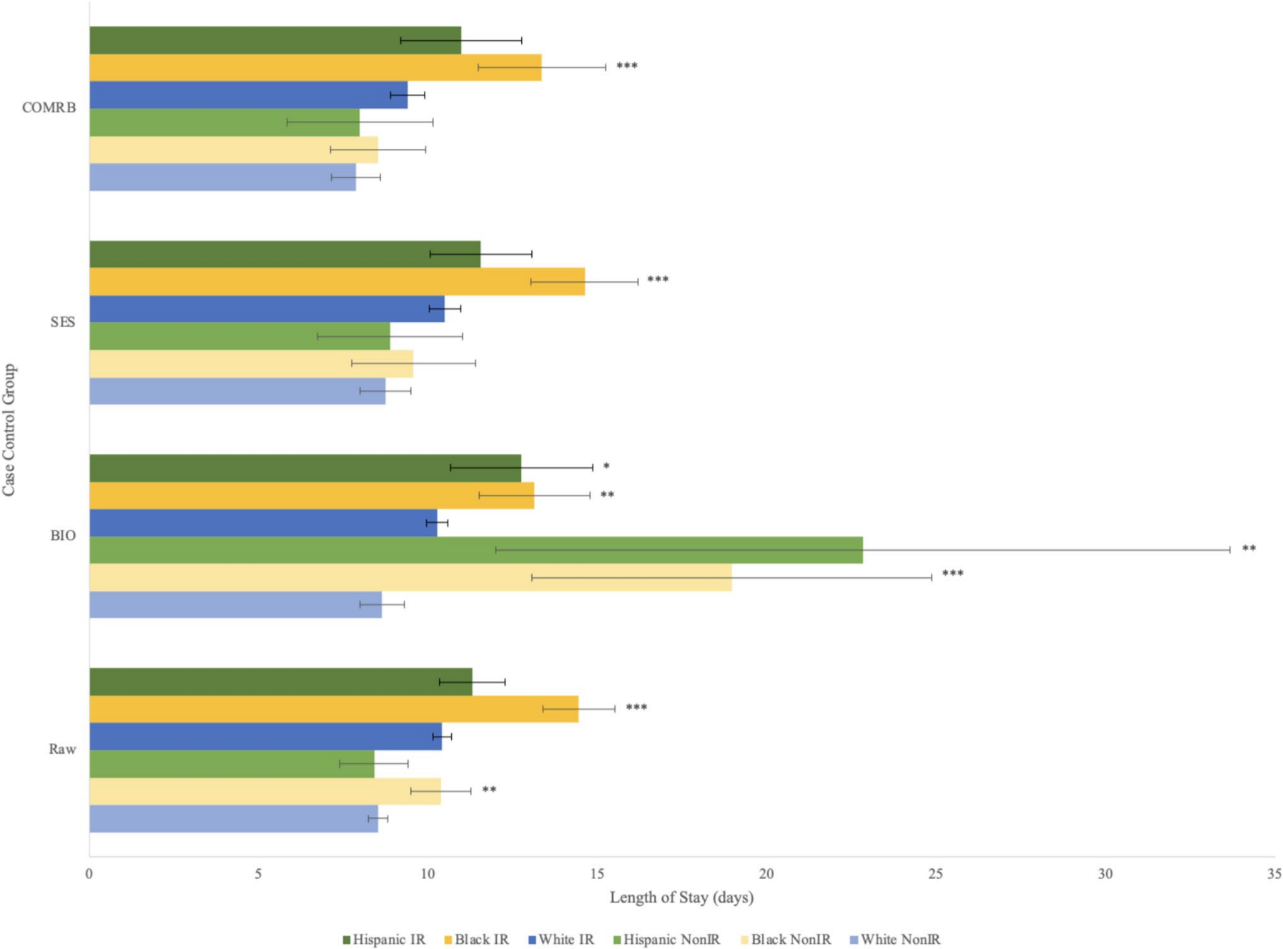
Length of stay by treatment type for case-controlled datasets 2016–2021. Length of stay by self-reported race for patients treated with either IR or non-IR treatment for acute upper non-variceal GI hemorrhage. Error bars indicate 95% confidence intervals. Significance between non-White race and White race of the same treatment type determined by two-tailed *t*-test. *** indicates *p* < 0.0001, ** indicates *p* < 0.001, * indicates *p* < 0.05. Significance markers shown between White race and non-White race for a given treatment type. Raw, uncontrolled dataset; BIO, controlled for age and biological sex; SES, controlled for age, biological sex, median zip code income quartile, and primary insurance type; COMRB, controlled for age, biological sex, and Charlson comorbidity index

NH Black patients had longer lengths of stay than White patients (12.61 vs 9.57; *p* < 0.0001, mean LOS in days). This was true for NH Black patients receiving IR treatment (14.46 vs 10.42; *p* < 0.0001) and those receiving non-IR treatment (10.38 vs 8.53; *p* < 0.001). Hispanic patients did not have a significantly different length of stay from White patients or NH Black patients (Fig. 1). LOS regardless of treatment type is shown for time subgroups in Fig. 1. Data comparing LOS for either IR or non-IR treatment is shown for 2016–2021 (Fig. 2).

Longer LOS persisted for NH Black patients compared to White patients in the matched groups BIO (13.78 vs 9.92), SES (12.94 vs 10.07), and COMRB (11.33 vs 8.88) (*p* < 0.0001). Longer LOS for IR treatment was the primary reason for these differences in SES (14.63 vs 10.49) and COMRB (13.35 vs 9.40) (*p* < 0.0001). LOS for non-IR treatment was not significantly different in these subgroups between races. To explain the longer LOS for NH Black patients receiving IR treatment, propensity score matching was done for two measures of bleed severity: number of blood transfusions and vasopressor use. However, longer LOS after IR treatment persisted for NH Black patients compared to White patients in the blood transfusion (14.46 vs 10.21) and vasopressor (14.43 vs 10.29) matched groups (*p* < 0.0001).

2020–2021 showed less difference in LOS based on race. No difference in LOS between races was seen in 2020–2021 for IR treatments when controlling for BIO, SES, and COMRB, whereas a difference was seen in those matched groups from 2016–2019 and 2016–2021. The same was seen for combined treatment types (Fig. 1) when controlling for SES and COMRB. There was no difference in LOS between races in 2020–2021 for non-IR treatment, contrasting 2016–2019 and 2016–2021. However, 2020–2021 did see an increased LOS for Hispanic patients compared to White patients receiving IR treatment, but this did not persist when controlling for BIO and SES.

## Discussion

There was no significant difference in likelihood of undergoing TAE for acute non-variceal upper GI hemorrhage based on race. Similarly, there was no significant difference in mortality odds based on race. These results contrast the inequities in IR utilization and mortality for treatments like TIPS in portal hypertension, uterine artery embolization for fibroids, AV fistula placement, and catheter-directed thrombolysis in pulmonary embolism [3, 5, 9, 10]. One possible explanation is that TAE for upper GI non-variceal hemorrhage is reserved for refractory cases and therefore the indications are more limited. The increased odds of mortality for IR treatment compared to non-IR treatment is expected considering that IR treatment is reserved for refractory cases, unstable patients who have failed to respond to medical and endoscopic management.

Length of stay is a common but imperfect gauge of morbidity. The literature is unclear on whether length of stay discrepancies between racial groups are common after IR procedures [5, 9]. It is also unclear whether specific comorbidities contribute to the increased length of stay. There was a significant difference in LOS between NH Black patients and White patients for both TAE and endoscopy, but the biological factors, SES, and chronic comorbidities were confounders for endoscopy; these confounders did not explain the LOS difference in IR treatment between NH Black and White patients. Bleed severity, measured by controlling for blood transfusions and vasopressor use, also did not explain the LOS differences.

The observed racial disparities in LOS for IR treatment may be explained by time to first intervention. For acute upper GI bleeds (variceal and non-variceal), early interventions have been shown to decrease length of stay, particularly when first endoscopy is done in under 24 h [11]. Black patients have also been shown to have longer emergency room wait times for GI bleeds, as well as longer times to endoscopy for variceal bleeding [12, 13]. Thus, though no data exist on Black patients’ wait times for TAE, extended wait times either pre-hospital or intra-hospital for Black patients could be the underlying cause of longer LOS for IR treatments. Pre-hospital factors could include distance from an IR-staffed hospital or distrust of the medical system; this is possible given only about 3000 interventional radiologists practice in the USA and are concentrated in certain areas [14]. However, this is less likely considering we controlled for condition severity via vasopressor use, thus favoring intra-hospital factors. Intra-hospital factors could include limited IR staffing/high patient volume, hospital communication practices or bleed guidelines, or provider bias (the NIS database unfortunately has no data on time to procedure, hospital county, or number of IR attendings). Thus, our data emphasize the need for future research to clarify the time from hospital arrival or onset of symptoms to the time of first intervention for NH Black patients. Future studies could also examine location of discharge, as placement issues in skilled nursing facilities may impact LOS—the NIS does not have discharge location data. Of note, NH Black patients’ LOS did not differ from White patients post-COVID-19, which may indicate changes in access after the pandemic.

While further research is done, practical clinical considerations for reducing NH Black patients’ LOS span the local and international levels. Physicians, particularly in IR, can be vigilant for barriers to care and morbidity risks in NH Black patients with acute non-variceal upper GI hemorrhage. Hospitals can assess whether their interdisciplinary communication, IR staffing, and discharge policies exacerbate or address this known disparity. Targeting the time to first procedural decision is also clinically reasonable. Loftus et al. showed the benefits of instituting multidisciplinary protocol for managing severe GI bleeds in the USA [15]. Time from admission to procedure, lengths of stay, and readmissions decreased for cases with a prompt initial conference call and follow-up between Interventional Radiology, Gastroenterology, and Acute Care Surgery. Such protocols could establish treatment decisions faster for NH Black patients and eliminate time-consuming paging and instant messaging. Unstable patients requiring immediate IR intervention could accordingly be taken to the IR suite faster and avoid worsening while teams struggle to communicate. Alternatively, for patients undergoing IR treatment after failed endoscopy, clinicians can target expediting time to first endoscopy so that refractory bleeds are identified sooner (i.e., shortening time to first IR procedure by shortening the time to prior endoscopy). Implementing this on a national or international level may require professional society action; the British Society of Gastroenterology’s (BSG) multi-society care bundle for upper GI hemorrhage offers a potential example of an effective intervention [16]. Their bundle standardizes upper GI hemorrhage care orders with time marks for actions that have been shown to expedite patient stabilization and gastroenterologist consultation, reducing LOS. However, both the Loftus and BSG approach have logistical challenges. Loftus’s model would require professional society recommendations akin to BSG’s to encourage widespread change in unstandardized health systems and change hospital on-call systems [17]. Meanwhile, the BSG care bundle lost participation over the course of the COVID-19 pandemic. Thus, these interventions need sustained emphasis to clinicians to work, but are practical clinical steps while more data are generated on NH Black patients’ time to IR treatment.

### Importance

This is the first work to examine disparities in acute nonvariceal upper GI hemorrhage by procedure. It adds important specificity for clinicians. Previous work showing disparities in incidence for upper GI hemorrhage may be unmotivating for proceduralists due to the broad array of risk factors, etiologies, and multiple specialties involved in this pathology. However, with this work on a narrower spectrum of upper GI hemorrhage and analyzed by procedure type, we offer valuable data to interdisciplinary teams of gastroenterologists, interventional radiologists, surgeons, and internists that make treatment decisions based on known data for specific procedures. We (1) show a disparity in treatment rather than incidence with serious implications for morbidity and cost and (2) clarify for proceduralists the disparities associated with their given procedure, adding clarity to where teams should concentrate their equity efforts and (3) establish a baseline to measure interventions’ efficacy against and (4) largely ruled out age, sex, SES status, and initial disease severity with our case control analysis so future work can be more targeted.

### Limitations

This study has limitations. The retrospective design is prone to bias and confounders, though propensity score matching mitigated this. The HCUP NIS also lacks data such as time to treatment that may explain LOS discrepancies, so a definite explanation of NH Black patients’ longer LOS cannot be offered. The Charlson comorbidity index was used because the relatively stronger Elixhauser comorbidity index was not included in HCUP NIS data before 2019. Furthermore, socioeconomic status was controlled for using surrogates; insurance type and median postal ZIP code income quartile may not reflect a specific patient’s condition. Finally, matching resulted in small sample sizes for NH Black and Hispanic patients from 2020 to 2021.

## Conclusion

In conclusion, there is no difference in acute upper GI non-variceal hemorrhage between White, NH Black, and Hispanic patients’ odds of receiving TAE or mortality odds. However, LOS is longer for NH Black patients. This is explained by confounders for non-IR treatment, but this difference persists in IR treatment even when controlling for age, sex, socioeconomic status, chronic comorbidities, and bleed severity. More data is needed on time to IR treatment. These findings may reinforce other studies that show clearly defined indications reduce disparities in procedures.

## Data Availability

The data supporting the findings of this manuscript are not available publicly due to the commercial sensitivity of the HCUP NIS. Researchers interested in the data may contact the authors with reasonable requests.
